# Super-resolution Microscopy Reveals Compartmentalization of Peroxisomal Membrane Proteins*[Fn FN2]

**DOI:** 10.1074/jbc.M116.734038

**Published:** 2016-06-16

**Authors:** Silvia Galiani, Dominic Waithe, Katharina Reglinski, Luis Daniel Cruz-Zaragoza, Esther Garcia, Mathias P. Clausen, Wolfgang Schliebs, Ralf Erdmann, Christian Eggeling

**Affiliations:** From the ‡ Medical Research Council Human Immunology Unit and; § Wolfson Imaging Centre, Weatherall Institute of Molecular Medicine, University of Oxford, Headley Way, Oxford OX3 9DS, United Kingdom,; ¶ Institute of Physiological Chemistry, Systemic Biochemistry, Ruhr University Bochum, Universitätsstrasse 150, 44801 Bochum, Germany, and; ‖ MEMPHYS-Center for Biomembrane Physics, Department of Physics, Chemistry and Pharmacy, University of Southern Denmark, Campusvej 55, 5230 Odense, Denmark

**Keywords:** membrane protein, membrane trafficking, microscopy, peroxisome, protein import, STED microscopy, super-resolution optical microscopy

## Abstract

Membrane-associated events during peroxisomal protein import processes play an essential role in peroxisome functionality. Many details of these processes are not known due to missing spatial resolution of technologies capable of investigating peroxisomes directly in the cell. Here, we present the use of super-resolution optical stimulated emission depletion microscopy to investigate with sub-60-nm resolution the heterogeneous spatial organization of the peroxisomal proteins PEX5, PEX14, and PEX11 around actively importing peroxisomes, showing distinct differences between these peroxins. Moreover, imported protein sterol carrier protein 2 (SCP2) occupies only a subregion of larger peroxisomes, highlighting the heterogeneous distribution of proteins even within the peroxisome. Finally, our data reveal subpopulations of peroxisomes showing only weak colocalization between PEX14 and PEX5 or PEX11 but at the same time a clear compartmentalized organization. This compartmentalization, which was less evident in cases of strong colocalization, indicates dynamic protein reorganization linked to changes occurring in the peroxisomes. Through the use of multicolor stimulated emission depletion microscopy, we have been able to characterize peroxisomes and their constituents to a yet unseen level of detail while maintaining a highly statistical approach, paving the way for equally complex biological studies in the future.

## Introduction

**FIGURE 1.**
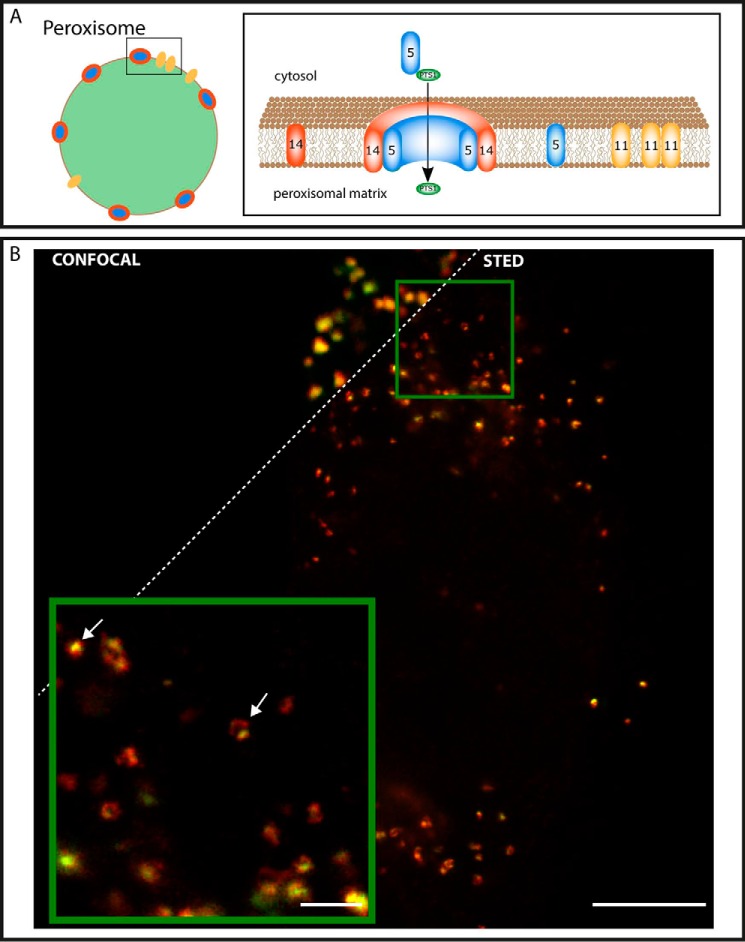
**STED imaging of peroxisomal membrane and matrix.** *A*, peroxisomal protein import process. A sketch of a peroxisome (*left*) and a close-up of a part of the peroxisomal membrane (*right*) with peroxisomal import receptor PEX5 (*blue*); membrane protein PEX14 (*orange*), both components of the translocation pore; PTS1 cargo protein (*green*); and proliferation factor PEX11 (*yellow*). The cargo receptor PEX5 binds PTS1-containing cargo proteins in the cytosol, directs them to the peroxisomal membrane where PEX5 becomes part of the translocation pore, the PTS1-containing cargo proteins become imported, and PEX5 is released afterward. *B*, representative dual color confocal (*upper left*) and STED (*lower right*) images of fixed human fibroblast cells transfected with the peroxisomal matrix marker GFP-SCP2 and immunostained for PEX14 (*red*; Abberior STAR 600 secondary antibody) and GFP-SCP2 (*green*; GFP nanobooster Abberior STAR 635P); overview (*main panel*) and zoom (*inset*; STED) of area marked in the overview. *Arrows*, examples of pointlike and ringlike SCP2 intensity patterns surrounded by PEX14, *i.e.* the peroxisomal membrane. *Scale bars*, 5 (*overview*) and 1 μm (*inset*).

Peroxisomes (see Fig. 1*A*) are small, membrane-enclosed organelles that fulfill many different functions of eukaryotic cells, including a variety of metabolic reactions (1). Peroxisomes contain a diverse collection of densely packed enzymes whose composition is largely dependent on the host organism, cell type, and tissue (2, 3). Peroxisomal function and protein composition, at a given time, are largely dependent on changes occurring in the external environment to which it responds dynamically (4). In human cells, peroxisomes are involved in the β-oxidation of fatty acids, reactive oxygen species detoxification, and biosynthesis of different lipids, including plasmalogen, the main component of the myelin sheath (5). Because of its crucial roles in human metabolism, defects in peroxisomal function can cause many severe diseases (6, 7). Only a fundamental understanding of peroxisomal biogenesis and assembly will help identify ways to control these diseases, leading to novel therapeutic strategies.

Most peroxisomal proteins, needed to maintain the diverse functions of the organelles, are encoded in the nucleus, synthesized in the cytosol, and imported post-translationally from the cytosol into the peroxisomes (8). These proteins are either inserted into the peroxisomal membrane or transported into the peroxisomal matrix. Translocation of folded and even oligomerized proteins across the peroxisomal membrane is a common principle, which differentiates peroxisomes from other organelles like mitochondria. Most proteins destined for the peroxisomal matrix carry a peroxisomal targeting signal, type 1 (PTS1),^5^ at their C terminus that is recognized by the cycling peroxisomal import receptor PEX5. After binding of the PTS1 cargo protein to PEX5 in the cytosol, this cargo-receptor complex is directed to the peroxisomal membrane where it interacts with the peroxisomal membrane protein PEX14. Here PEX5 is integrated into the membrane, and it forms a transient translocation pore together with PEX14 (9, 10) (see Fig. 1*A*). Upon cargo translocation, membrane-associated PEX5 is monoubiquitinated at the cytosolic side of the membrane. This modification functions as an export signal, resulting in the ATP-dependent removal of PEX5 from the peroxisomal membrane (11, 12). The released PEX5 is available for another round of protein import. Although recent studies shed light on function and mechanisms of the translocation of peroxisomal matrix proteins across the peroxisomal membrane, many details of the organization of the peroxisomal translocon mainly consisting of PEX14 and PEX5 are not yet known due to missing spatial resolution in applied standard optical microscopy techniques. To overcome this limitation, we implemented a multicolor super-resolution stimulated emission depletion (STED) (13) microscope setup that allowed this investigation to be performed with sub-60-nm resolution (14).

Besides the import complex, represented by PEX14, membrane-bound PEX5, and GFP-sterol carrier protein 2 (SCP2), a PTS1 cargo protein, we included two further proteins to form a contrast and control to the peroxisomal import system, the peroxisomal proliferation factor PEX11β and the mitochondrial protein TOM20. PEX11β was used as an internal peroxisomal control as it is supposed to be independent from the peroxisomal import machinery and one of the most abundant integral membrane proteins not involved in protein import. PEX11β facilitates the extension of the membrane during the peroxisomal fission process and therefore plays an essential role in this process as well as in its initialization (15). We will simply refer to PEX11β as PEX11 throughout this text. TOM20, a part of the translocation complex of the outer mitochondrial membrane, represented an external control as it is localized in an unrelated organelle. Furthermore, recent super-resolution studies have been performed on TOM20 localization in the mitochondria (16) and so this protein also added a control in terms of the quality of our nanoscale imaging and fixation methods while remaining suitability detached from the peroxisomes to highlight the similarities between the stains of peroxisomal proteins.

An increased spatial resolution for imaging peroxisomes within cells should for the first time allow studying the protein distribution on the peroxisomal membrane. Possible heterogeneity in these distributions in between peroxisomes may on one hand highlight yet undetected molecular rearrangements at the membrane linked to peroxisome aging, proliferation, or differences in the uptake of peroxisomal matrix proteins and most importantly to peroxisomal malfunction. On the other hand, these imaging and analysis techniques could easily be transferred to studying other small cellular organelles such as endosomes or lysosomes.

This work introduces the first detailed visualization of peroxisomal membrane proteins with subdiffraction resolution in mammalian cells. Through STED microscopy, we show that peroxisomes are diverse in their appearance and that no individual visualization or qualitative description could accurately represent their heterogeneity. To solve this issue, we adopted quantitative imaging and analysis to approach the diverse characteristics of the peroxisomes. Starting with an optimized multicolor STED imaging protocol, we established a quantitative analysis pipeline involving careful calibration and corrections to ensure that super-resolution was achieved and confounding artifacts were corrected. The result of this effort has been an accurate and thorough characterization of the morphology of peroxisomes by visualization of different peroxisomal proteins. We specifically found a subpopulation of peroxisomes in which the membrane proteins PEX5 and PEX14 as well as PEX11 and PEX14 are separated in distinct compartments on the peroxisomal membrane, resulting in lower colocalization of these proteins. Our data highlight the power of STED microscopy but also the need for experimental optimization and custom data analysis for revealing novel details of the spatial organization of proteins at the peroxisomal membrane.

## Results

### 

#### 

##### Visualization of Peroxisomal Matrix and Membrane Proteins via Dual Color STED Microscopy

To investigate the morphology and size of peroxisomes, we started by imaging the peroxisomal membrane marker PEX14 and peroxisomal matrix marker SCP2 in fixed human fibroblasts (GM5756T). We used a primary antibody in combination with a secondary fluorescently labeled (Abberior STAR 600) antibody for immunolabeling PEX14 and transfection with GFP-SCP2 in combination with Abberior STAR 635P GFP nanobooster for labeling SCP2 and analyzed their distribution on a two-color STED microscope (using 594 and 635 nm laser light for fluorescence excitation and 755 nm as STED laser light (supplemental Fig. 1)). When imaged with diffraction-limited confocal microscopy (*i.e.* without the STED laser light), peroxisomes appear to be stained homogeneously by both matrix and membrane markers. However, with the STED microscope, enabling a lateral resolution below 60 nm for both signals, a clear separation of peroxisomal membrane and matrix proteins becomes evident for most of the observed peroxisomes, revealing a broad distribution of heterogeneous features with variation in size from a minimum of 130 nm to a maximum of 650 nm in diameter (Fig. 1*B*). Line profiles across typical images of a single peroxisome (PEX14 staining) displayed a full width at half-maximum of 330 ± 125 nm (mean ± S.D.; *n* = 100). In some cases, the SCP2 can be found concentrated in round shaped clusters surrounded by the peroxisomal membrane, indicated by the PEX14 staining. In other cases, PEX14 forms larger, ringlike structures. Here, the SCP2 does not seem to be in the center of the peroxisomes but confined to the periphery of the organelle, attached to the membrane.

##### Distribution of PEX5, PEX14, PEX11, and TOM20 around the Peroxisomal Matrix Marker SCP2 via Single Color STED Imaging

**FIGURE 2.**
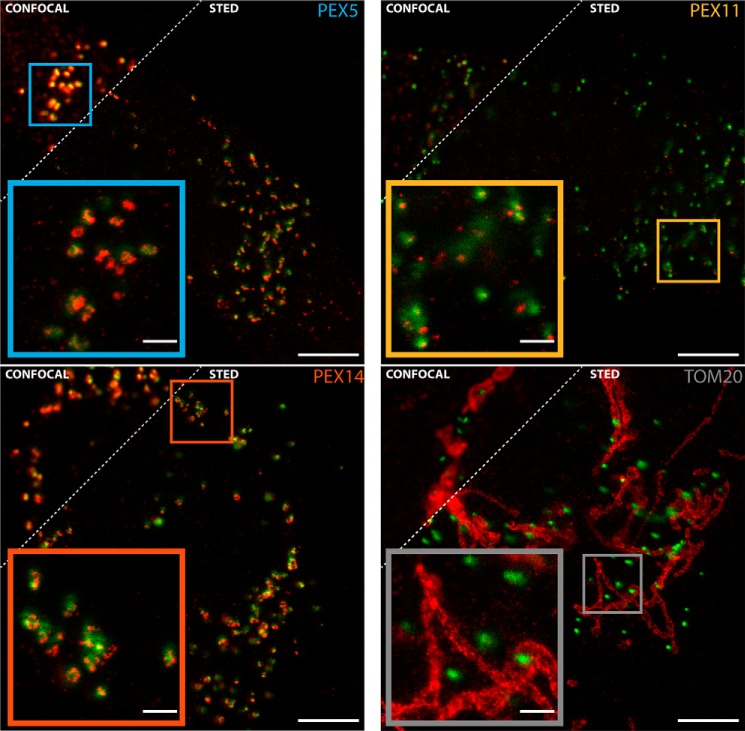
**STED imaging of selected peroxisomal and mitochondrial membrane proteins.** Representative dual color confocal (*upper left*) and STED (*lower right*) images of fixed human fibroblast cells transfected with the peroxisomal matrix marker GFP-SCP2 (*green*; always confocal) and immunostained (*red*) for PEX5 (*upper left panel*), PEX14 (*lower left panel*), PEX11 (*upper right panel*), and TOM20 (*lower right panel*), overviews (*main panels*), and zooms (*insets*; STED) of areas marked in the overviews are shown. *Scale bars*, 5 (*overviews*) and 1 μm (*insets*).

For further investigations on the heterogeneity in protein organization at peroxisomes, the GFP-SCP2 was used as a reference marker to identify peroxisomes processing an active import of PTS1 proteins (note that SCP2 is a PTS1 protein). Only cells with a bright expression and peroxisomal localization of the GFP-SCP2, indicated by the characteristic punctate pattern of peroxisomal staining, were used for further analysis (Fig. 2). The GFP-SCP2 signal was acquired in the confocal modality, and the spatial distribution of the peroxisomal proteins (peroxins) PEX14, PEX5, and PEX11,and the mitochondrial outer membrane protein TOM20 in the area of the identified peroxisomes was studied with the STED microscope. PEX14 and PEX11 reside in the membrane of peroxisomes, whereas the receptor PEX5 does so only transiently, switching between cytoplasmic and membrane-bound states as it shuttles PTS1-containing proteins into the peroxisome (Fig. 1*A*). For studying membrane-bound PEX5, only an antibody specifically recognizing the membrane-bound conformation (17) was used.

Fig. 2 shows representative images of fixed fibroblast cells with peroxisomal localization of GFP-SCP2 and immunostained for either PEX5, PEX14, PEX11, or TOM20 (see “Experimental Procedures” for details). STED images show that the peroxins PEX5, PEX14, and PEX11 are located predominantly in regions containing GFP-SCP2, and only the TOM20 distribution is clearly distinguishable from the SCP2 location. In addition, the super-resolved images revealed that the analyzed peroxins are not homogeneously distributed across the peroxisomal membrane but have characteristic spatial organizations. Although PEX5 and PEX14 show a broad distribution of features from small circular blobs to bigger ringlike or elliptical structures, PEX11 is organized in smaller, more roundish features. Conversely and as expected, TOM20 staining is localized to the mitochondrial network and is, broadly distributed all over the cell.

**FIGURE 3.**
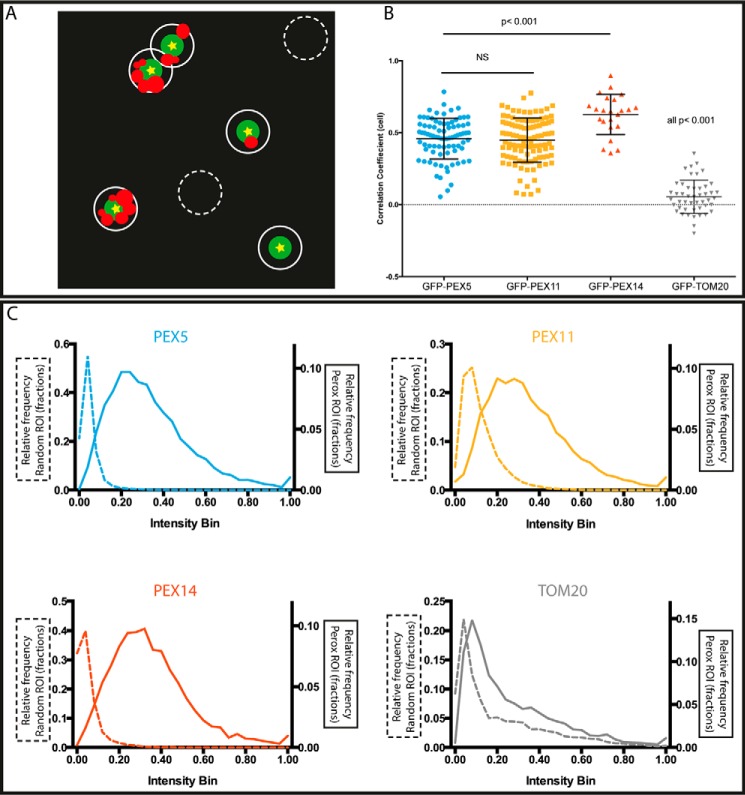
**Intensity analysis of protein distributions at peroxisomes.** *A*, scheme of automated algorithm for defining peroxisomal (*Perox*) (*white circles*) and random non-peroxisomal (*dashed white circles*) regions of interest (*ROI*). Only peroxisomes with GFP-SCP2 signal, *i.e.* active protein import, were chosen. For the analysis, circular patches of 190-nm radius centered at the maximal intensity (*yellow stars*) of the confocal GFP-SCP2 signal (*green*) were chosen for peroxisomal regions and patches enclosing no GFP-SCP2 signal for random regions and the detected intensity of the antibody staining within these regions were analyzed. *B*, intensity correlation analysis of GFP-SCP2 and antibody staining signal (for different proteins PEX5, PEX11, PEX14, and TOM20 as labeled); values of 1 indicate maximum correlation, and 0 indicates no correlation (*dashed horizontal line*). Shown are individual values from 82 (PEX5), 107 (PEX11), 23 (PEX14), and 47 (TOM20) selected peroxisomes (average and S.D. (*error bars*); *horizontal bars*, *p* test results (*NS*, non-significant)). *C*, histograms of the normalized intensity distribution of PEX5 (*top left*), PEX11 (*top right*), PEX14 (*bottom left*), and TOM20 (*bottom right*) in the peroxisomal (*full lines*) and random (*dashed lines*) regions.

To study this system in such a way that the heterogeneity is properly represented, a detailed imaging acquisition protocol and an elaborate quantitative image analysis were applied. The peroxisome locations were defined as the intensity maxima appearing in the GFP-SCP2 signal (note that the most accurate determination of these maxima was provided by recording the GFP signal in a confocal mode, *i.e.* without the use of nanoboosters or the STED super-resolution option). Subsequent analysis was then applied to highlight the characteristics of the protein staining in circular regions of 380 nm in diameter around 1) the identified peroxisomes matrix marker and 2) in randomly selected regions, as a control, away from the GFP-SCP2 signal (Fig. 3*A*).

##### Intensity Correlation Analysis

We first investigated how the fluorescence intensity detected for the peroxins and TOM20 correlates with that of the GFP-SCP2 staining. For this, the correlation coefficient was calculated from the paired intensities around each peroxisome (within the 380-nm-diameter circle) and averaged over each cell. The distribution of these values is shown in Fig. 3*B*. The GFP signal was found to be consistent across all analyzed conditions, which indicates equal levels of SCP2, *i.e.* equal PTS1 protein uptake across all peroxisomes, at least those that actively participated in protein import. PEX5, PEX11, and PEX14 show positive intensity correlations with the GFP signal, whereas TOM20 shows hardly any correlation. Upon close inspection, PEX14 shows a significantly (*p* < 0.001) higher intensity correlation (0.63 ± 0.14, mean ± S.D.; 1 = maximum correlation, 0 = no correlation) compared with PEX11 (0.45 ± 0.15) and PEX5 (0.45 ± 0.14). This might reveal the tighter link of PEX14 to the import of PTS1 proteins. Despite the difference, the correlation coefficient of the GFP-SCP2 signal with the antibody staining of the different peroxins is above 0.4 for all three membrane-bound peroxins, indicating an overall strong relationship between imported SCP2 and the amount of peroxisomal membrane proteins. Mitochondrially bound TOM20 in contrast showed only a very low intensity correlation with SCP2, indicating a weak or no relationship between PTS1 import and the amount of TOM20 at close proximity to the peroxisomes.

##### Intensity Distribution Analysis

We next more deeply hunted possible differences in the abundance of the different peroxisomal proteins at the peroxisomes by analyzing the frequency distribution of the intensity of the observed signal. As before, using STED microscopy on the same samples, we determined the overall intensity of the immunostained peroxins in each identified peroxisomal regions (selection as before; Fig. 3*A*) and normalized it to the maximum intensity for each cell, resulting in values between 0 and 1. The frequency histograms of these relative intensity values (Fig. 3*C*, *bold lines*) confirm that all the peroxisomes contain measureable amounts of peroxisomal membrane proteins PEX5, PEX11, and PEX14. The intensity frequency histograms of the latter are very alike with very similar peak and standard deviation values (when approximated by a Gaussian distribution 0.28 ± 0.17 for PEX5, 0.30 ± 0.18 for PEX11, and 0.31 ± 0.16 for PEX14). Moreover, because there are no zero intensity peaks in our data, it shows that peroxisomes actively importing the GFP-SCP2 proteins never lack one of the three peroxins. Furthermore, the relative intensity of the TOM20 staining close to the actively importing peroxisomes is significantly lower (peak at 0.11) than for the peroxins, indicating that most peroxisomes show low staining of TOM20 relative to the background. The same intensity distribution analysis was repeated in randomly chosen regions that do not include any GFP-SCP2 signal (Fig. 3*C*, *dotted lines*). In this control case, all histograms show a sharp peak close to zero (peak values <0.1) with a significant number of zero intensity events. For the membrane-associated peroxins, this evidence shows an expected clear difference in abundance near and far away from the peroxisomes. In contrast, the intensity distribution of TOM20 is very similar between peroxisomal and the non-peroxisomal regions. However, the statistically relevant higher peak value in the case of peroxisomal proximity (0.11 *versus* 0.08) indicates a slightly higher abundance of TOM20 close to the peroxisomes.

##### Morphological Analysis

**FIGURE 4.**
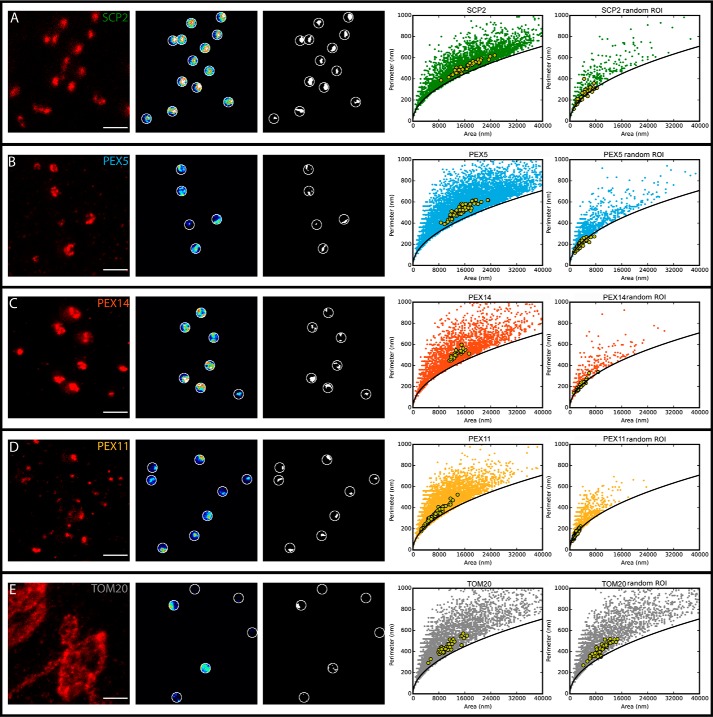
**Morphology analysis of protein distributions.** A morphology study of GFP-SCP2 (*A*), PEX14 (*B*), PEX5 (*C*), PEX11 (*D*) and TOM20 (*E*) is shown. *Left panels*, representative image of the antibody staining in STED resolution (*scale bar*, 1 μm); *second panels from left*, intensity distribution of the antibody staining in selected peroxisomal regions; *third panels from left*, thresholded features of distributions of antibody staining around peroxisomes as the basis for the morphology analysis; *right panels*, scatter plot of value pairs of area and perimeter of the selected features for all peroxisomes (*filled circles*; 13,759 (GFP-SCP2), 9,058 (PEX5), 9,784 (PEX11), 2,570 (PEX14), and 4,699 (TOM20) peroxisomes) and for cell-averaged values (*green circles*; 111 (GFP-SCP2), 82 (PEX5), 107 (PEX11), 23 (PEX14), and 47 (TOM20) cells). *Solid lines* depict expected dependence for circular features. *ROI*, region of interest.

Qualitative visual assessment of the STED images of protein distributions at the peroxisomes revealed a great diversity in appearance. As highlighted before, PEX11 is organized in smaller, more roundish features, whereas the staining patterns for PEX5 and PEX14 are more heterogeneous, ranging from small circular blobs to bigger ringlike or elliptical structures. To quantify this diversity, a morphology analysis was performed on the STED images (Fig. 4, *left panels*) taken for immunolabeled PEX5, PEX11, PEX14, and TOM20 as well as for SCP2 (nanobooster). As before, circular regions around each individual peroxisome were identified by the GFP-SCP2 signal. Each selected circular patch (Fig. 4, *second panels from left*) was thresholded, and the resulting binary mask (or structural features; Fig. 4, *middle panels*) was evaluated for its morphological properties. A suitable metric for evaluation of these structures was the comparison of perimeter and area. This approach allows the broad heterogeneity of cluster shapes to be visualized in a relatively low dimensional space in the form of a scatter plot of paired values (perimeter and area) where each *dot* represents one peroxisome within the population of cells (*i.e.* depicting variability over all peroxisomes) and the larger *green circles* represent values averaged over all peroxisomes within one cell (*i.e.* cell-to-cell variability) (Fig. 4, *right panels*). The *black line* in the scatter plots provides orientation and represents the perimeter-area dependence that a perfectly circular structure would show at the different scales. Spherical structures are expected to lie close to this line, whereas tubular and ringlike structures possess much greater perimeter values than expected for a circular area; *i.e.* the scatter point lies above the *black line*. The spread in the value pairs statistically confirms the wide range of sizes and shapes in protein organization at the peroxisomes. However, the morphology analysis now allows us to characterize differences between the proteins that become most evident on the cell-averaged values (*larger green circles*). Both PEX5 and PEX14 show a broad distribution in size; however, the morphology diverges from a circular shape (*i.e.* the value pairs divert from the *black line* representing circular shape). A detailed analysis indicates that the morphology of PEX5 varies more strongly than does that PEX14 and that PEX5 clusters are slightly (*p* < 0.001) larger on average than PEX14 clusters. This becomes obvious when comparing the population average and S.D. for the morphological parameters for both proteins (average cluster area, 14.9 ± 3.1 (PEX5) and 13.0 ± 1.7 nm^2^ (PEX14); perimeter length, 510 ± 65 (PEX5) and 485 ± 50 nm (PEX14)). In contrast, PEX11 is slightly less heterogeneously distributed around a peroxisome, displaying smaller, more circular shapes with an average area of 5.4 ± 2.3 nm^2^ and perimeter of 265 ± 80 nm (*p* < 0.001 *versus* PEX5 and PEX14). Conversely, randomly chosen regions away from the peroxisomes show morphologies characterized by very small and circular patterns (Fig. 4, *far right panels*), indicating that the organization of the proteins is distinct at the peroxisomes. SCP2 staining shows a broad distribution in size, however, mainly of round shaped features, indicated by the positioning of the value pairs close to the *black line*. The average area of the SCP2 staining patterns is 18.2 ± 4.1 nm^2^ and the perimeter is 530 ± 60 nm, indicating that on average the SCP2 patterns are larger and more roundish than those of PEX5 and PEX14 but similarly heterogeneous in size. The spatial patterning of TOM20 is very similar at peroxisomal and in randomly selected regions but forms slightly larger structures (9.0 ± 2.8 *versus* 6.3 ± 2.0 nm^2^) with perimeters (370 ± 80 *versus* 270 ± 60 nm) at the peroxisomes, which confirms the weak correlation of the mitochondrial protein with peroxisomal positions.

##### Colocalization Studies Using Dual Color STED Microscopy

**FIGURE 5.**
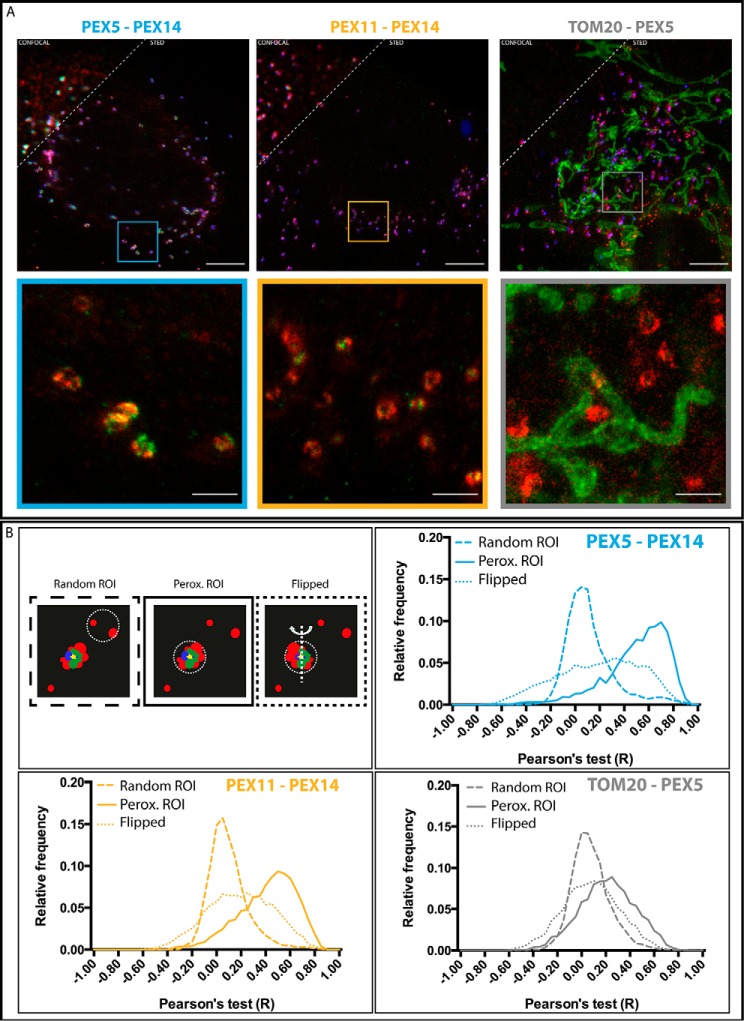
**Colocalization study of proteins at peroxisomes.** *A*, representative confocal (*upper left*) and STED (*lower right*) images of fibroblast cells transfected with the peroxisomal matrix marker GFP-SCP2 (*blue*; only confocal and only in *top panels*), fixed, and immunolabeled for PEX14 (*red*) and PEX5 (*green*) (*left*), PEX14 (*red*) and PEX11 (*green*) (*middle*), and TOM20 (*green*) and PEX5 (*red*) (*right*). Overviews (*upper panels*) and zooms (*insets*) of regions marked in overviews are shown. *Scale bars*, 5 (*overviews*) and 1 μm (*insets*). *B*, Pearson's test colocalization analysis of different peroxisomal proteins. *Upper left panel*, scheme of the analysis procedure. Circular patches surrounding GFP-SCP2 signal (peroxisomal regions of interest (*ROI*)) and non-GFP-SCP2 signal (random regions of interest) were selected, and colocalization values were calculated using a pixel-wise Pearson's test. *Upper right* and *lower panels*, frequency histogram of Pearson's test values (−1, opposing colocalization; 0, no colocalization; 1, maximum colocalization) for PEX5 *versus* PEX14 (*upper right*), PEX11 *versus* PEX14 (*lower left*), and TOM20 *versus* PEX5 (*lower right*) and for random regions of interest (*dashed lines*), peroxisomal (*Perox*) regions of interest (*solid lines*), and flipped (*dotted lines*) (number of data points: PEX5-PEX14, 5439; PEX11-PEX14, 6178; TOM20-PEX5, 4305).

Through using dual color STED imaging, we were able to relate the spatial staining of more than one protein simultaneously with a 60-nm spatial resolution. Fig. 5*A* shows representative dual color STED images from our study on peroxisomal proteins. We analyzed three different conditions, each a different pairing of immunolabeled protein, in fixed fibroblasts: PEX5 *versus* PEX14, PEX11 *versus* PEX14, and TOM20 *versus* PEX5. As before, the signal of the GFP-SCP2 was additionally recorded in confocal mode as a means of identifying actively importing peroxisomes. We analyzed more than 30 images for each condition acquired on at least 10 different cells from at least three separate samples. Our analysis compares the spatial distribution of the proteins within each peroxisomal region first by performing a pixel-wise Pearson's colocalization test to quantify the colocalization of PEX5/PEX14, PEX11/PEX14, and TOM20/PEX5 (values of 1 indicate complete colocalization, 0 indicates no colocalization, and −1 indicates opposing colocalization). Fig. 5*B* shows the histogram of colocalization values over the entire peroxisome population (*solid line*). PEX5/PEX14 show the highest colocalization with a median Pearson's value of 0.55 compared with 0.45 for PEX11/PEX14 and 0.22 for TOM20/PEX5.

**FIGURE 6.**
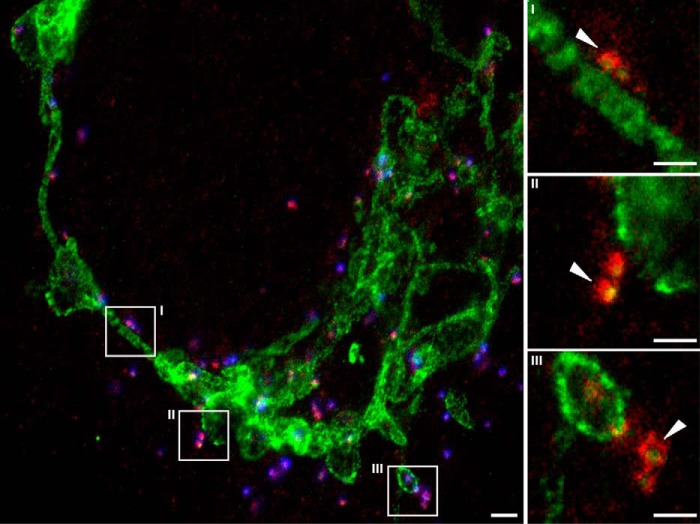
**Examples of colocalization of PEX5 and TOM20.** A representative STED image of human fibroblasts transfected with GFP-SCP2 (*blue*), fixed, and immunolabeled for PEX5 (*red*) and TOM20 (*green*) is shown. Also shown are an overview image (*left panel*) and zooms *I–III* (*right panels*) of the respective highlighted areas in the overview images, visualizing events of colocalization between PEX5 and TOM20 (*white arrows*). *Scale bars*, 2 (*overview*) and 0.5 μm (*zooms*).

Two different controls were applied for the colocalization analysis, a “random region” control (Fig. 5*B*, *dashed line*) and a “flip” control (Fig. 5*B*, *dotted line*). The first random region control was similar to the previous cases (Fig. 5*B*, *upper left panel*; compare with Fig. 3*A*) with the colocalization of the staining of the respective two proteins being analyzed in random locations away from the peroxisomes. All protein combinations show similarly low colocalization in these randomly chosen regions. In the second flip control, the colocalization of the staining of the respective two proteins is calculated within the peroxisomal regions after mirroring (or flipping) the spatial signal distribution along the vertical axis in one of the channels (Fig. 5*B*, *upper left panel*). This flip control is used to check the level of colocalization, which coincidentally arises due to the dense concentration of the proteins within the peroxisomal regions. However, in all cases, the protein pairs hardly showed any colocalization after this flip, indicating that the bulk of observed colocalization is a reflection of congruent compartmentalized protein distributions and does not result from a general homogeneous distribution around the peroxisomes. Our data thus highlight strong colocalization of all proteins within compartments at the peroxisomes with the highest level for PEX5/PEX14, slightly less for PEX11/PEX14, and the lowest level for TOM20/PEX5. However, the colocalization of TOM20/PEX5 is also statistically higher at the peroxisomes than at random regions, once again highlighting the slightly increased abundance of TOM20 at the peroxisomes, which seems to be correlated with the spatial organization of PEX5, which is also obvious from visual inspection of the images (Fig. 6).

##### Compartmentalization Analysis by Dual Color STED Microscopy

**FIGURE 7.**
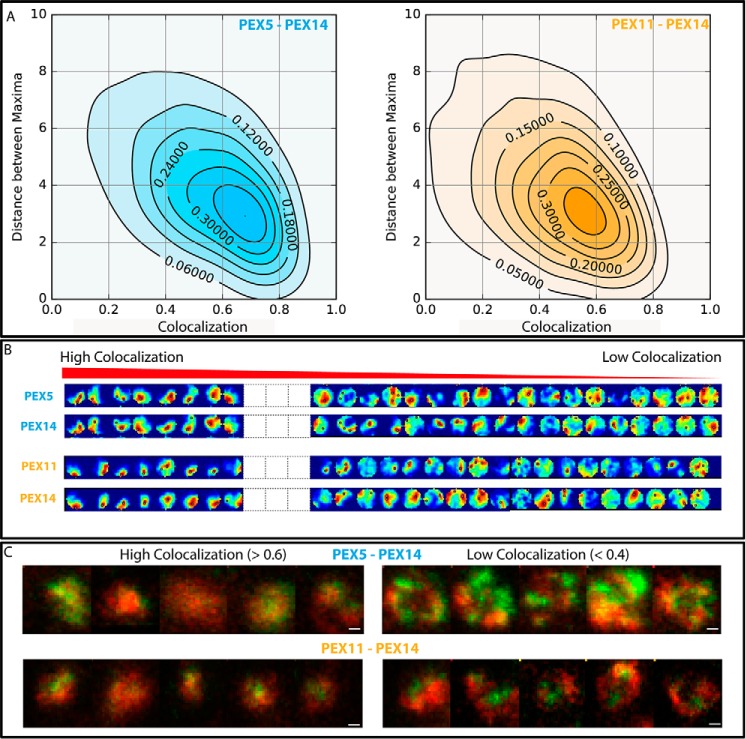
**Compartmentalization of peroxisomal membrane proteins.** *A*, anticorrelation between compartmentalization and colocalization of PEX5 *versus* PEX14 (*left*) and PEX11 *versus* PEX14 (*right*). Normalized frequency plots of value pairs of average nearest distance between maxima within the intensity distribution (as a measure of compartmentalization) and Pearson's colocalization test from single peroxisomes (5439 for PEX5-PEX14 and 6178 for PEX11-PEX14) are shown. *B*, representative patterns of the intensity distribution in the circular regions around single peroxisomes from PEX5 and PEX14 (*upper two panels*) and PEX11 and PEX14 (*lower two panels*) derived from dual color STED images and ordered from the highest to the lowest Pearson's test colocalization value (*left* to *right* as labeled). Only the extreme cases of high and low colocalization are shown; medium cases are left out (*white boxes*; for full sequences see supplemental Fig. 2). *C*, representative dual color STED images of PEX5 (*green*) and PEX14 (*red*) (*upper panels*) as well as PEX11 (*green*) and PEX14 (*red*) (*lower panels*) for strong colocalization (Pearson's test values >0.6) and low compartmentalization of both proteins (*left panels*) and for low colocalization (Pearson's test values <0.4) and high compartmentalization of both proteins (*right panels*). *Scale bars*, 200 nm.

To further highlight the compartmentalized spatial distribution of the proteins at the peroxisomal membrane, we developed a more rigorous image analysis regime to examine the trends in the data. This resulted from close inspection of the two-color STED images where although there was a tendency toward a compartmentalized distribution of the proteins at the same time there was also an indication that there was a significant number of peroxisomes with a low level of colocalization, for example PEX5 and PEX14 or PEX11 and PEX14 (Fig. 5*A*). This is quantitatively shown by a fraction of peroxisomes exhibiting a Pearson's coefficient that tended toward zero (Fig. 5*B*). To confirm this tendency, we expanded our colocalization analysis. As mentioned, the spatial intensity distribution of each protein staining at a single peroxisome is usually strongly compartmentalized and characterized by multiple maxima. We determined the locations of the maxima within the intensity distribution of each protein staining. We plotted the average interchannel distance between those maxima for a region against the corresponding Pearson's correlation value both for PEX5/PEX14 and PEX11/PEX14, resulting in a reciprocal relationship between both parameters. These data show that large distances between maxima (or strongly compartmentalized protein distributions) are characterized by a low colocalization with other proteins (Fig. 7 and supplemental Fig. 2). Representative protein distribution patterns with high and low Pearson's correlation values are depicted in Fig. 7*C*. Peroxisomes characterized by high colocalization between PEX5 and PEX14 or PEX11 and PEX14 appear more circular and less compartmentalized compared with the low colocalization cases where multiple maxima are present. Furthermore, note the slightly stronger colocalization between PEX5 and PEX14 compared with PEX11 and PEX14; however, both comparisons still show the same tendency between colocalization and compartmentalization.

## Discussion

Our super-resolution STED imaging of immunostained peroxisomes in fixed fibroblasts revealed that peroxisomes are small, predominantly circular shaped organelles with a heterogeneous size and shape distribution. From our analysis, we show that peroxisomes vary between around 130 and 650 nm in diameter, indicating that subperoxisomal details like protein distribution and proximity cannot be studied with standard optical microscopy but require super-resolution microscopy.

Our study is focused on the investigation of the peroxisomal import translocon, represented by the PTS1 import receptor PEX5 and its interaction partner on the peroxisomal membrane, PEX14. We studied the distribution of PEX5 and PEX14 relative to the characteristically punctate peroxisomal matrix marker SCP2, an indicator of actively importing peroxisomes. Moreover, we investigated the peroxisomal proliferation factor PEX11, which has no known role in protein import. The distribution of the mitochondrial protein TOM20 was studied as a non-peroxisomal control.

In the first part of our studies, the super-resolved (<60-nm resolution) STED images disclosed that the matrix marker SCP2 is not equally distributed in the center of the peroxisomes in all cases. It sometimes shows a clear shift to one side of the peroxisome and seems to stick to the membrane (Fig. 1*B*). Moreover, the actively importing peroxisomes have a broad range of shape and sizes, even within one cell, and furthermore peroxisomal membrane proteins are not equally distributed over the surface of peroxisomes but are located in distinct domains (Fig. 2). To study this phenomenon, we optimized our image acquisition as well as analysis to further characterize this heterogeneity, including a large number of acquired data (more than 30 independent images for each condition) and custom designed analysis routines to quantitatively highlight the diverse and global characteristics of peroxisomal membrane proteins.

Single color STED imaging in combination with intensity correlation analysis revealed a strongly positive correlation of the intensity of staining for PEX5 and PEX14 at the peroxisomes to that of the peroxisomal matrix marker GFP-SCP2 (Fig. 3*B*). This mainly reveals a positive dependence between the amount of peroxisomal protein import and the abundance of PEX5 and PEX11 at the peroxisomal membrane. A statistically larger correlation of PEX14 with SCP2 suggests that PEX14 is an indicator of the import activity of peroxisomes and might be the limiting factor for this process. This would follow from the known role of PEX14 as an integral part of the import translocon pore. The slightly lower correlation between SCP2 and PEX5 is most probably a consequence of the transient localization of PEX5 at the peroxisomal membrane.

An analysis of the heterogeneity in protein staining at each peroxisome (Fig. 3*C*) highlights that all actively importing peroxisomes contain PEX5 and PEX14; *i.e.* there is no population of those peroxisomes lacking one of the two analyzed proteins involved in the formation of the PTS1 translocon. A detailed analysis of the morphology of the PEX5 and PEX14 staining patterns (Fig. 4) reveals a strong heterogeneity in the shape and size around peroxisomes for both, ranging from small globular blobs to larger elliptical and ringlike structures. However, the morphological distribution of staining patterns is very alike for membrane-bound PEX5 and PEX14 because PEX5 is integrated into the peroxisomal membrane to form the translocon together with PEX14. However, the distribution of patterns showed slightly more cell-to-cell variability in the case of PEX5 compared with PEX14, indicating a greater variability in the peroxisomal localization of PEX5, potentially reflecting a cell-wide regulation. The morphology analysis also revealed that the PEX5 clusters are slightly larger than those of PEX14. This might possibly represent PEX5 inserting into the membrane during the docking/insertion process as epitope detection by the antibody used is only possible after conformational alterations of PEX5 that are supposed to be induced upon contact with peroxisomal membranes or proteins.

To further explore the fine detail of PEX5 and PEX14 localization, we conducted a dual color STED imaging approach and a subsequent colocalization analysis (Fig. 5), which again highlighted a strong heterogeneity. Most peroxisomes showed a strong colocalization of both proteins, which is to be expected from their obvious interaction at the translocon (18–20). However, our analysis also depicted subpopulations of peroxisomes showing only a weak colocalization between PEX5 and PEX14. This could represent stages in which PEX14 is interacting either with PEX19 (21) or microtubules (22). These both compete with PEX5 as they all bind to the same binding site of PEX14. Nevertheless, to investigate these subpopulations in more detail, the morphological distribution of peroxins was compared with their colocalization (Fig. 7 and supplemental Fig. 2). Cases of strong colocalization are characterized by roundish and small staining patterns at the peroxisomal membrane, whereas weak colocalization is characterized by larger ringlike or elliptical staining patterns of both PEX5 and PEX14, highlighting clear compartmentalized organization. The differences do not show up as two distinct peroxisome populations, but rather the protein patterning covers the whole range between both extremes. We have to note that the variation of the staining patterns in size and shape between ringlike and dotted features does not follow from imaging peroxisomes at different axial planes (*e.g.* at the equatorial *versus* basal plane) because such artificial heterogeneity should correlate with total intensity of the patterns (dotted patterns should show lower intensities), which we did not observe.

In the case of the peroxisomal proliferation factor PEX11, our STED microscopy analysis also revealed that PEX11 is located at all actively importing peroxisomes, showing their capability to proliferate, and that PEX11 is not specifically recruited to peroxisomes to initiate proliferation. This is further supported by the fact that the amount of PEX11 is correlated with the amount of imported protein. Although not confirmed by quantitative data, this correlation might follow from an increased size of the peroxisome; *i.e.* the larger a peroxisome becomes, the more PEX11 it contains, opening up the speculation that a certain amount of PEX11 might trigger the implementation of peroxisomal fission to maintain peroxisomal size. In contrast to PEX5 and PEX14, the morphology of PEX11 is clearly different, being much smaller and more round. To our knowledge, this is the first high resolution analysis of endogenous PEX11 compartmentalization in human cells. In previous studies, often modifications of PEX11, like GFP-PEX11β fusion proteins, were used under overexpression conditions (23, 24). It is known that PEX11 interacts with itself and forms oligomers (25–28), most likely leading to the formation of roundish PEX11 clusters at the peroxisomal membrane. Whether these small clusters of PEX11 are starting points for membrane extensions for peroxisomal proliferation cannot be justified from our data. Such starting points are thought to be tubular extensions, which are still too small to be visualized with a spatial resolution of 60 nm, or these regions might be excluded from our analysis as they do not contain matrix proteins like GFP-SCP2 (24, 29, 30). Moreover, cells containing predominantly tubulated peroxisomes, a known change in peroxisomal morphology prior to fission (31), were excluded from the analysis to concentrate on one population of peroxisomes only, and the investigation of peroxisomal fission with super-resolution microscopy will be a challenging task for future investigations; our current study was rather focused on the peroxisomal translocon. Nevertheless, PEX11 shows a high colocalization with PEX14, indicating an association between PEX11 and PEX14, *i.e.* a localization of PEX14 close to the smaller PEX11 domains. Conversely, there is a huge amount of PEX14 staining in areas not containing any PEX11.

**FIGURE 8.**
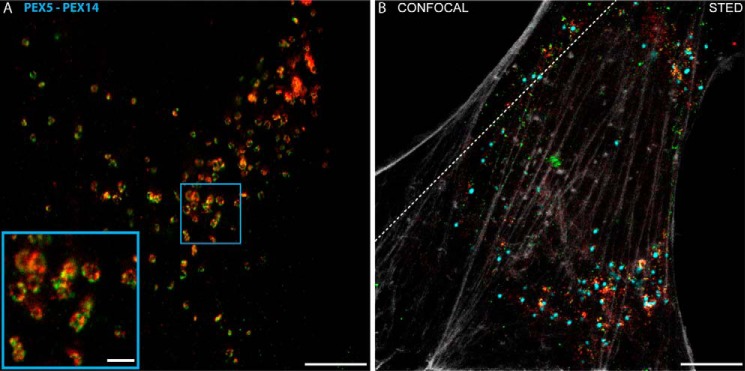
*A*, example of synchronized appearance of ringlike PEX5-PEX14 intensity distribution patterns around single peroxisomes. A representative dual color STED image of human fibroblasts fixed and immunolabeled for the PEX5 (*green*) and PEX14 (*red*), overview image (*main panel*), and zoom (*inset*) of the marked area depicting the ringlike patterns are shown. *Scale bars*, 5 (*overview*) and 1 μm (*inset*). *B*, four-color imaging. Representative confocal (*upper left corner*) and STED (*lower right*) images of fixed human fibroblast cells transfected with GFP-SCP2 (*cyan*; always confocal mode), fixed, and immunolabeled for PEX5 (*green*), PEX14 (*red*), and β-actin (*gray*) are shown. *Scale bar*, 5 μm.

As mentioned, the staining patterns for PEX5 and PEX14 at the peroxisomal membrane vary from concentrated round features to elliptical, ringlike structures. In some cells, the formation of the latter ringlike structures appears synchronized as if the formation of this morphology is triggered by an unknown signal (Fig. 8*A*). The physiological relevance of these different morphologies remains speculative. One possible explanation may be a membrane remodeling shortly before the induction of the fission process. However, PEX11 should be involved in this progress, but the morphology of this protein gives no hint as to the formation of membrane extensions, the initial step of peroxisomal proliferation. Another possibility may be a difference in the uptake of peroxisomal matrix proteins through the peroxisomal translocon. Here a separation of PEX14 and PEX5 at the membrane may indicate a less active import, possibly caused by maturation of the peroxisomes, such as a decrease in import activity in older peroxisomes. However, a decreased import may also be present in peroxisomes of aging cells, representing a known decrease in peroxisomal protein import in such cells (32). We have to note that the variation between ringlike and dotted features cannot be explained.

Using the mitochondrial protein TOM20 as a non-peroxisomal control in all the analyses, we found a slightly but statistically significant higher abundance of TOM20 at the peroxisomal membrane compared with random non-peroxisomal regions. Functional association and contact sites between mitochondria and peroxisomes have been described (33, 34). However, whether our observations represent those possible contact sites between peroxisomes and mitochondria or a mistargeting of TOM20 to peroxisomes remains to be elucidated.

Although the presented data are descriptive and do not give a functional explanation of the observed phenomena, we describe for the first time the organization of different peroxisomal membrane proteins. This method can help to understand remodeling of the membrane-associated proteins during peroxisomal protein import, maturation, and fission as well as at malfunctioning peroxisomes, which will be the topic of future investigations. However, the outlined procedures also provide tools for investigating protein organization at other small organelles such as endosomes or lysosomes.

In future studies, we intend to optimize super-resolution STED imaging on peroxisomes by implementing further colors, *i.e.* being able to correlate the positions of even more proteins in super-resolution mode (as highlighted for actin in Fig. 8*B*), and by using STED microscopy with improved spatial resolution along all three spatial dimensions (35). Nevertheless, through using multicolor STED microscopy, we have been able to characterize peroxisomes and their constituents to a so far unprecedented level of detail while maintaining a highly statistical approach, paving the way for equally complex biological studies in the future.

## Experimental Procedures

### Sample Preparation

Human fibroblasts (GM5756T; Moser, Baltimore, MD) were maintained in a culture medium consisting of DMEM with 4500 mg of glucose/liter and 110 mg of sodium pyruvate/liter supplemented with 10% fetal calf serum, 2 mm glutamine, and 1% penicillin-streptomycin. The cells were cultured at 37 °C in 5% CO_2_. Cells were grown on Number 1.5 cover slides and transfected with peGFP-SCP2 (36) using Lipofectamine 2000 transfection reagent (Invitrogen). 24 h after transfection, the cells were fixed with 3% formaldehyde in PBS, permeabilized for 6 min with pure methanol at −20 °C, and blocked with 2% BSA + 5% FCS in PBS for 1 h at room temperature. Permeabilization with methanol was used as Triton X-100 is known to extract PEX11 from the peroxisomal membrane (37). Samples were incubated with primary antibodies in blocking buffer for 1 h at room temperature. To stain peroxisomal proteins, antibodies recognizing PEX14 (38), PEX5 (17), and PEX11B (Abcam, Cambridge, UK) were used. Mitochondria were visualized using an antibody against TOM20 (FL-145, Santa Cruz Biotechnology, Dallas, TX). After several washing steps, the cells were incubated for 30 min with secondary antibodies conjugated to Abberior STAR 600 and/or Abberior STAR Red (Abberior Instruments, Goettingen, Germany) diluted 1:250 in 1% BSA in PBS. To perform super-resolution imaging also on the GFP-SCP2 protein, a GFP nanobooster conjugated to Abberior STAR 635P (ChromoTek GmbH, Planegg-Martinsried, Germany) diluted 1:200 was used. To perform four-color imaging, phalloidin-ATTO490LS (Sigma-Aldrich) was used to label actin filaments. After several washing steps, the slides were mounted on a drop of Mowiol (Sigma-Aldrich). We tested and compared different immunolabeling and fixation protocols to minimize artifacts due to unspecific binding or fixation/immunolabeling-induced changes in protein distribution.

Supplemental Fig. 1 depicts the use of fluorescent crimson beads. Fluorescent microspheres (FluoSpheres; crimson (625/645); diameter, 0.02 μm; Invitrogen) were diluted 1:10^4^ in Milli-Q water, a drop of the diluted beads was attached to the coverslip using poly-l-lysine (Sigma-Aldrich), and finally the coverslip was mounted on a microscope slide and embedded in the mounting medium Mowiol (Sigma-Aldrich).

For nuclear pore immunolabeling (supplemental Fig. 1), human fibroblast cells (GM5756T) were fixed with 3% paraformaldehyde for 10 min, rinsed several times in PBS, permeabilized in 0.1% Triton X-100 for 10 min, rinsed again several times in PBS, and finally blocked in 2% BSA + 5% FCS in PBS for 1 h. Samples were incubated with primary mouse antibodies to stain nuclear pore complexes (Abcam) diluted 1:500 in blocking buffer for 1 h at room temperature. Coverslips were washed five times in 1% BSA in PBS and then incubated for 30 min with goat anti-mouse antibodies Abberior STAR 600, Abberior STAR Red, and ATTO490LS diluted 1:250 in blocking buffer. Samples were washed five times in 1% BSA in PBS and mounted in Mowiol.

### STED Setup

Supplemental Fig. 1 depicts the scheme and the performance of the STED microscope. The multicolor STED microscope was realized by coupling a titanium-sapphire STED laser (MaiTai HP, Spectra-Newport) into an Abberior Instruments Resolft system (14). This system has been configured to work with three pulsed excitation lasers (640, 594, and 485 nm; LDH-D-C-640P and LDH-D-C-485P, Picoquant, Berlin, Germany, and PDL 594, Abberior Instruments). This adaptation included (i) the spatial overlay of the three excitation and STED lasers using a main dichroic mirror (ZT740SPRDC, AHF Analysentechnik, Tuebingen, Germany) and a sufficient amount of mirrors to control the beam pathways (at least two in each separate beam), (ii) the temporal synchronization of the laser pulses by triggering the excitation laser diodes with the STED laser using a photodiode (APS-100-01, Becker and Hickl, Berlin, Germany), (iii) the shuttering and adjusting of the power of the STED laser with an acousto-optical modulator (MT110-B50A1.5-IRHK, Photon Lines, Banbury, UK), (iv) the stretching of the STED laser pulses using two 20-cm-long glass rods (SF6 glass) and a 120-m-long single mode polarization-maintaining fiber (AMS Technologies/OZ Optics), (v) the incorporation of a vortex phase plate (VPP-1a, RPC Photonics, Rochester, NY) into the collimated STED beam after the fiber realizing the doughnut-shaped intensity distribution at the focus, and (vi) the exact control of the circular polarization of the STED laser by introducing a half-wave plate and a quarter-wave plate along the optical path of the STED beam (B. Halle, Berlin, Germany). To ensure stability of the overlay and perfect polarization and to achieve zero local doughnut intensity, we utilized careful detection of the laser polarization (SK010PA-VIS, Schaefter + Kirchhoff, Hamburg, Germany), stable mirror holders, polarization-maintaining single mode fibers, and mirrors and polarization optics with λ/10 surface flatness (especially dichroic mirrors with a 5-mm substrate (ZT740SPRDC, AHF Analysentechnik)) to maintain the laser wave fronts. The excitation and STED beams were coupled into a 100 ×/1.4 numerical aperture oil immersion objective lens (UPlanSApo 100×/1.4 oil, Olympus, Japan). To precisely set the time alignment between laser pulses the microscope's FPGA-PC card was utilized. For scanning the excitation and STED beams, the beam scanning unit of the Abberior system was exploited. The movement along the *z* axis was controlled by a piezo stage (MIPOS100, Piezosystem Jena GmbH, Jena, Germany). To detect and separate the fluorescence signal, three different avalanche photodiodes and three different color filters were utilized (APD1, 500–570 nm; APD3, 605–625 nm; APD2, 650–730 nm). All acquisition operations were controlled by the microscope's Imspector software (Abberior Instruments).

#### 

##### Imaging

The custom built microscope guaranteed the imaging of up to three different super-resolved signals (STED laser, 755 nm) optimized for the fluorescent labels Abberior STAR 600 (excitation, 594 nm; detection, APD3), Abberior STAR RED (excitation, 635 nm; detection, APD2), and ATTO490LS (excitation, 488 nm; detection, APD2) and one confocal channel to detect GFP emission (excitation, 488 nm; detection, APD1). A total of four overlapping images (three STED and one confocal) can be acquired, making it a flexible instrument to determine spatial distributions and proximities of multiple molecules at subdiffraction detail (Fig. 8*B*).

By selecting a 755-nm doughnut intensity distribution of the focused STED laser, this microscope provided a lateral resolution below 60 nm for imaging of dyes such as Abberior STAR RED and Abberior STAR 600 and a lateral resolution under 80 nm for the long Stokes shift dye ATTO490LS. We experienced negligible cross-talk (<5%) between the fluorescence collected confocally in APD1 (GFP signal) and in APD2 (Abberior STAR RED or ATTO490LS) or APD1 and APD3 (Abberior STAR 600). Consequently, the confocal and STED recordings were acquired in parallel. Cross-talk between the STED recordings in APD2 and APD3 were minimized by subsequently recording the images for each label (line-by-line or frame-by-frame alteration). Spatial drift occurring during these serial acquisitions was corrected for by using the repeatedly recorded GFP-SCP2 confocal signal (APD1) as a reference.

The biggest advantage of this microscope, intrinsic to its design (39), was that no chromatic aberration occurred between the three super-resolved signals as only one doughnut-shaped STED laser was applied, which defined the position of the residual fluorescence of all labels. The only chromatic correction that remained to consider was between the GFP confocal signal (no STED beam on) and the super-resolution signals. To overcome this problem, the necessary chromatic correction was calculated through visualization and registration of TetraSpeck beads (0.1-μm TetraSpeck microspheres, Life Technologies) imaged under the same imaging regime as the experimental samples.

Only cells transfected with GFP-SCP2 showing a punctate peroxisomal pattern and normal shape were chosen for imaging. For each selected cell, an area of 30 × 30 μm was detected at a fixed pixel size of 20 nm and a fixed pixel dwell time of 40 μs. Excitation powers were measured at the back aperture of the objective lens: 35 microwatts for 485 nm, 45 microwatts for 594 nm, and 20 microwatts for 640 nm. To provide the same spatial resolution for Abberior STAR RED and Abberior STAR 600, the 755 nm STED laser powers were adapted to 80 and 150 milliwatts (back aperture of the objective lens) for the two dyes, respectively.

##### Image Analysis

Raw images were processed to correct for chromatic shift between the STED and confocal channels, and two-color STED images were also corrected for any drift occurring during their acquisition. The magnitude of the chromatic correction applied was estimated through visualization and registration of TetraSpeck beads imaged under the same imaging regime as the experimental samples. Drift correction was estimated through repetitive imaging of the GFP-SCP2 signal and was only necessary when two or more wavelength channels were acquired under the STED regime. The displacement between the first and second acquired GFP-SCP2 images was calculated through translation of one of the input images relative to the other using a 18 × 18-pixel search window (the drift was observed to not be greater than this margin). At each translation point, the product of the two images was calculated, and the overall minimum product was taken to represent the best registration of the two images. Through registration of the acquired GFP-SCP2 images, it was then possible to correct the STED images with a high degree of accuracy as these were acquired simultaneously to the GFP-SCP2 channels.

##### Maximum Finding Algorithm

Individual peroxisome fragments were identified from the GFP-SCP2 channel using the Fiji/ImageJ “Find Maxima” algorithm on a Gaussian smoothed image (σ = 2.0). Detection of maxima was kept consistent throughout using a noise tolerance parameter of “10.” A circular region (diameter, 19 pixels; 380 nm) was then superimposed on each detected location, and all of the regions were saved for subsequent analysis. For every detected region, a random location was also generated to sample areas where GFP-SCP2 staining and thus peroxisomes were not likely to be present. This was achieved by randomly translating each of the detected regions to a different point within a 180-pixel radius of the original location but constrained so as not to pick an existing region, which might contain another fragment of GFP-SCP2 fluorescence. This method was effective at finding random regions that were close to the peroxisome fragments but not overlapping and so ensured accurate comparisons between peroxisome-containing and non-peroxisome regions. These randomly perturbed regions were saved and used for subsequent comparisons as with the original set.

##### Correlation Analysis and Coefficient Calculation

Correlation analysis was performed on the raw pixel data in each of the previously detected regions (Fig. 2*B*). The intensity contained within each region was first integrated, in the GFP-SCP2 and in the STED channel under comparison, to form each measurement pair (*g_i_*, *r_i_*), and then the Pearson's correlation coefficient was calculated from all the measurements (*I*) in each cell.

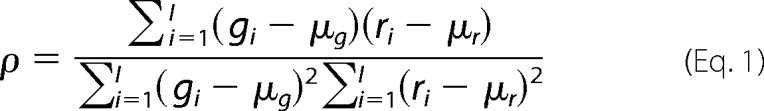
 where μ*_g_* and μ*_r_* are equal to the mean intensity of all the summed patches in the cell. To compare the mean values of correlation from each condition, one-way analysis of variance was performed with Tukey's pairwise comparison to test for a statistical difference between the comparisons.

##### Intensity Analysis

To establish the intensity distribution profile of each channel, a histogram was created over the integrated intensity values of the peroxisomal (*g_i_*) and random (*g*^rand^*_i_*) regions *i*. For each cell, the individual measurements in both sets were normalized (*g_i_*/max(*g_i_*), *g*^rand^*_i_*/max(*g_i_*)) with the maximum integrated intensity (max(*g_i_*)) within the cell and then binned using a bin size of 0.04, representing 25 bins between 0.0 and 1.0. The histogram for each condition was then accumulated by pooling all of the normalized values from all cells.

##### Morphology Analysis

To establish the shape distribution of peroxin stainings and the nanoboosted GFP signal, a morphological analysis was performed. In each detected peroxisomal and complementary random region, the intensity values were isolated and thresholded using the Fiji/ImageJ “MaxEntropy” method. The resulting segmented binary mask was then despeckled and eroded one iteration to produce an accurate representation of the bright cluster staining in each region. Each cluster was then analyzed, comparing its perimeter (pixels around the edge) and its area (total pixels). Each cluster was then plotted, comparing perimeter and area. Clusters that are perfectly circular will have a close relationship, linking their perimeter to the circumference of a circle, π*d* (shown by the curve superimposed on each graph; Fig. 4). Particles that are non-spherical, tubular, or fragmented will have a perimeter >π*d* where *d* is the diameter of the circle.

##### Pearson's Value Colocalization

To compare the spatial similarities of the peroxin stains in the STED resolution images, a pixel-based colocalization analysis was performed. For each detected region, the individual pixel values *j* were correlated using the Pearson's test.

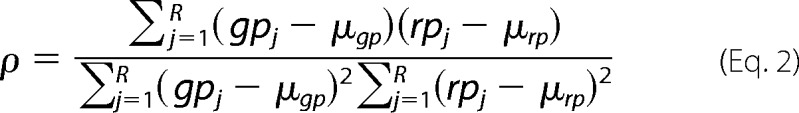
 where *gp_j_* and *rp_j_* represent the individual pixel values for the first and second detection channels, respectively, and μ*_gp_* and μ*_rp_* represent the average intensities over the region in the respective channel. Data from each region of every cell were collated, and a histogram was generated over the data. The analysis was repeated for the randomly positioned regions and using the peroxisomal regions but with the spatial arrangement of the input pixels flipped in the second of the two channels. The purpose of the flip was to test how high the colocalization value would be as a result of chance/coincidence colocalization, a possible consequence of dense protein packing within the peroxisome.

##### Compartmentalization Analysis

Having found the colocalization coefficient for each region, we were interested in establishing how different levels of colocalization corresponded to different spatial arrangements of the fluorescence in each region. To do this, we smoothed the individual regions (σ = 1.0) and then used a maximum finding algorithm to locate the maxima in the intensity distribution of each channel. This analysis was performed using Python scripting language and custom written functions. First, a maximum filter was applied to a smoothed input region, and the output of this was stored. The stored output was then compared pixel by pixel with the input region, and the matching values were saved. These stored pixel locations, which were the same value in the input patch and the output maximum filter, represented the local maxima. These detected sets of local maxima were then refined, only those maxima within 50% of the global maxima were retained, and any regions that were very flat (*e.g.* all zero values) were also excluded. From these locations of maxima, we then measured the distance to the nearest maxima in the complementary channel, recording the interchannel average distance for all the peaks in the region. These data were then plotted, comparing the colocalization values for each patch region against the average distance between the nearest maxima.

## Author Contributions

S. G. conducted most of the experimental work and microscopy development. D. W. developed and performed the data analysis. K. R. helped with the experimental work. L. D. C.-Z. helped in optimizing protocols. E. G. helped in optimization of the immunolabeling. M. P. C. helped with experimental setup. C. E., S. G., D. W., K. R., W. S., and R. E. designed research. C. E., S. G., D. W., and K. R. wrote the manuscript. All authors commented on the manuscript and research.

## Supplementary Material

Supplemental Data

## Footnotes

5 The abbreviations used are:

PTS1: peroxisomal targeting signal, type 1
STED: stimulated emission depletion
SCP2: sterol carrier protein 2.
