# Supplementary material for: Super-resolution Microscopy Reveals Compartmentalization of Peroxisomal Membrane Proteins
Source: J Biol Chem. 2016 Jun 16;291(33):16948–62. doi: 10.1074/jbc.M116.734038 (PMC5016101; doi:10.1074/jbc.M116.734038)
Supplement: Supplemental Data [file supp_291_33_16948__index.html]

Super-resolution Microscopy Reveals Compartmentalization of Peroxisomal Membrane Proteins — Super-resolution STED Microscopy of Peroxisomes — Supplemental Data 

# Super-resolution Microscopy Reveals Compartmentalization of Peroxisomal Membrane Proteins

## Supplemental Data

**Files in this Data Supplement:**

- Supplemental Figures - STED setup and compartmentalisation analysis
