# Supplementary material for: Super-resolution Microscopy Reveals Compartmentalization of Peroxisomal Membrane Proteins
Source: J Biol Chem. 2016 Jun 16;291(33):16948–62. doi: 10.1074/jbc.M116.734038 (PMC5016101; doi:10.1074/jbc.M116.734038)
Supplement: Supplemental Data [file supp_M116.734038_Supplemental_revision.pdf]

Super resolution microscopy reveals compartmentalization of peroxisomal membrane proteins

Silvia Galiani[1,*], Dominic Waithe[2,*], Katharina Reglinski[1], Luis Daniel Cruz-Zaragoza[3], Esther Garcia[2], Mathias P. Clausen[1,4], Wolfgang Schliebs[3], Ralf Erdmann[3], Christian Eggeling[1]

Supplementary Figure 1

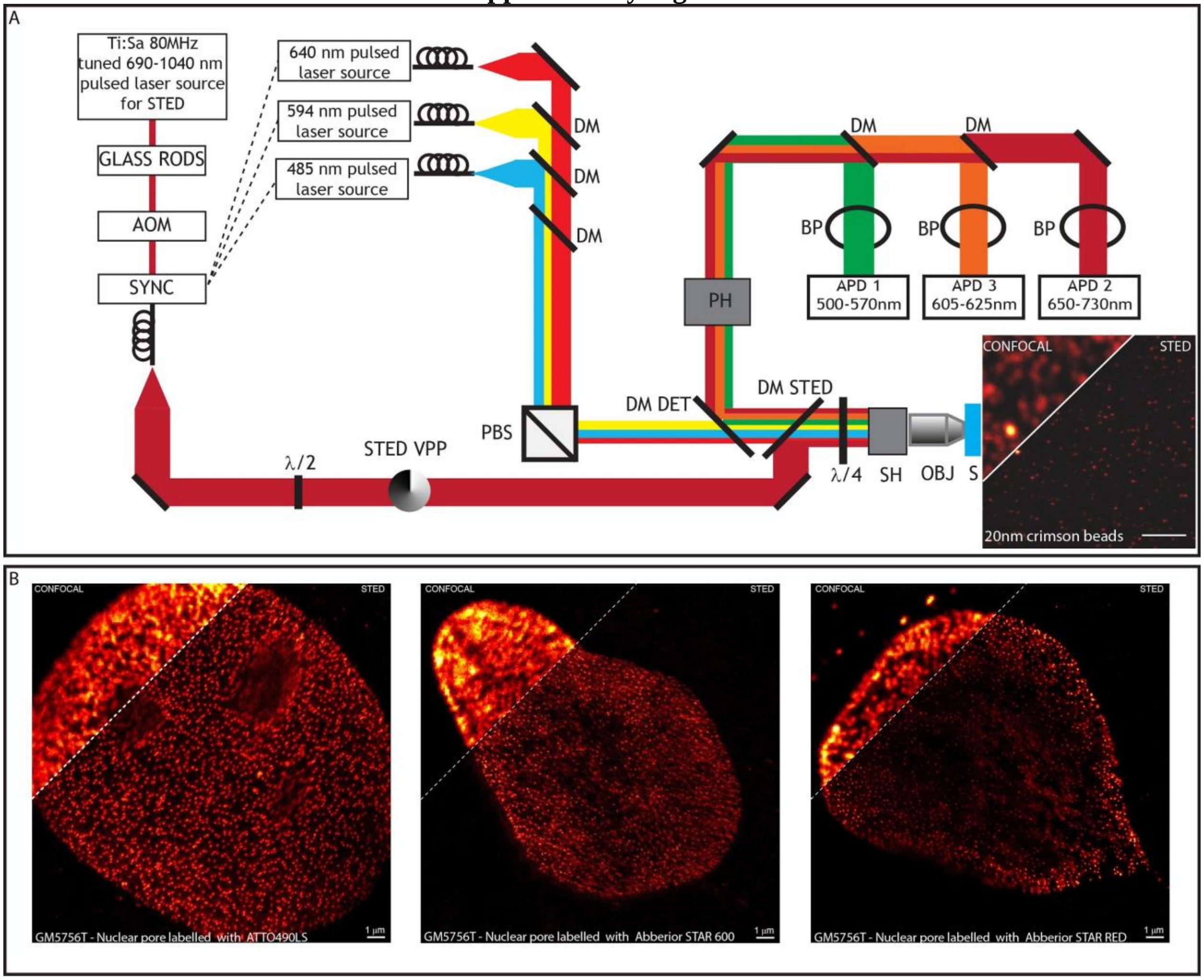

Supplementary Figure 1. *STED microscope setup and performance.* (**A**) Scheme of the STED microscope setup for multi-colour confocal and super-resolution imaging. AOM: acousto optic modulator; SYNC: synchronization signal; PBS: polarizing beam splitter; VPP: vortex phase plate; DM: dichroic mirror; BP: band pass filter; SH: scanning head; OBJ: objective lens; S: sample. (Right panel) Confocal (upper left) and STED (lower right) images of 20 nm fluorescent crimson beads, highlighting the improvement in spatial resolution with STED. (**B**) Confocal (upper left) and STED (lower right) images of nuclear pore complexes in fixed human fibroblast cells, immunolabelled with ATTO490LS (left), Abberior STAR 600 (middle) and Abberior STAR RED (right); excitation at 485 nm, 594 nm and 640 nm, and detection at 500-570 nm (APD1), 605-625 nm (APD3) and 650-730 nm (APD2), respectively, and STED laser at 755 nm. Scale bars 1 µm.

Supplementary Figure 2

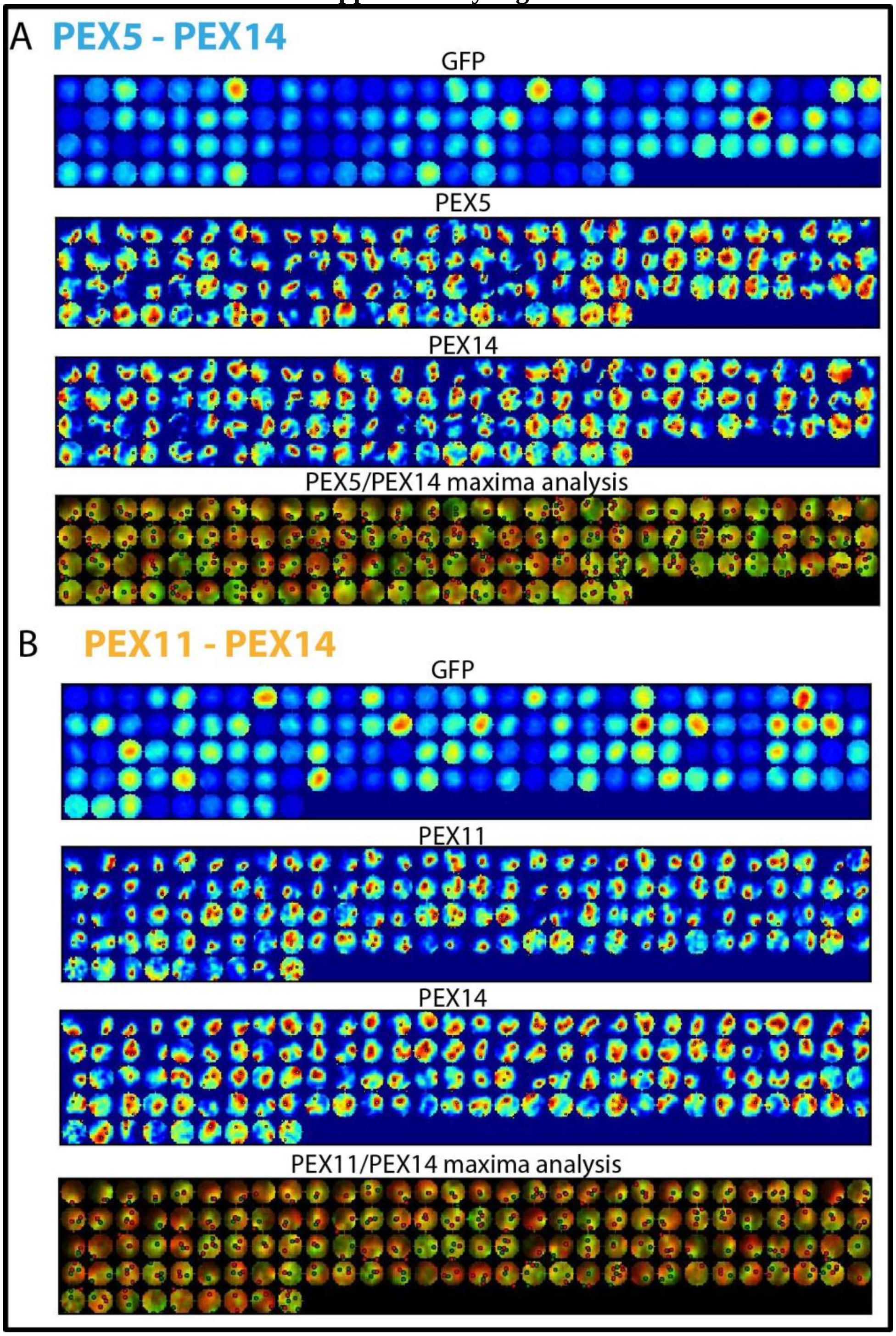

Supplementary Figure 2. *Further examples of anti-correlation between compartmentalization and colocalization of (**A**) PEX5 vs PEX14 and (**B**) PEX11 vs PEX14 (compare Fig. 6).* Representative patterns of the intensity distribution in the circular regions around single peroxisomes for GFP-SCP2 (first row), PEX5 in A and PEX11 in B (second row) and PEX14 (third row) from single-colour STED images, and results from maximum intensity positions analysis for PEX11-PEX14 from dual-colour STED images (fourth row; intensity plotted in green (PEX5 in A and PEX11 in B) or red (PEX14) with black dots indicating maximum intensity positions). The regions are ordered from highest to the lowest Pearson’s test

colocalization value (left to right and top to bottom), revealing a linear relationship between the distance of the maxima in intensity distribution (i.e. increased compartmentalization) and the Pearson's test colocalization value, i.e. strong compartmentalization correlates to a low colocalization between the two respective proteins (see also Figure 6).
